# Theoretical virtues in eighteenth-century debates on animal cognition

**DOI:** 10.1007/s40656-020-00332-z

**Published:** 2020-08-10

**Authors:** Hein van den Berg

**Affiliations:** Department of Philosophy, Institute for Logic, Language and Computation, University of Amsterdam, Oude Turfmarkt 143, Room 0.12, 1012 GC Amsterdam, The Netherlands

**Keywords:** Animal cognition, Theoretical virtues, Mechanism, Buffon, Reimarus, Condillac, Leroy

## Abstract

Within eighteenth-century debates on animal cognition we can distinguish at least three main theoretical positions: (i) Buffon’s mechanism, (ii) Reimarus’ theory of instincts, and (iii) the sensationalism of Condillac and Leroy. In this paper, I adopt a philosophical perspective on this debate and argue that in order to fully understand the justification Buffon, Reimarus, Condillac, and Leroy gave for their respective theories, we must pay special attention to the theoretical virtues these naturalists alluded to while justifying their position. These theoretical virtues have received little to no attention in the literature on eighteenth-century animal cognition, but figure prominently in the justification of the mechanist, instinctive, and sensationalist theories of animal behavior. Through my philosophical study of the role of theoretical virtues in eighteenth-century debates on animal cognition, we obtain a deeper understanding of how theoretical virtues were conceptualized in eighteenth-century science and how they influenced the justification of theories of animal cognition.

## Introduction

As Robert Richards has shown, there are at least three main theoretical positions within eighteenth-century debates on animal cognition: (i) Buffon’s mechanism, which provides mechanical explanations of animal behavior, (ii) Reimarus’ theory of instincts, which posits innate instincts to explain animal behavior, and (iii) the sensationalism of Condillac and Leroy, a mentalist position which attributes reason or intelligence to animals (Richards 1979, 1987). There are many possible historical explanations for why these different positions emerged: we can point to different sociological reasons, differences in the intellectual context within which Buffon, Reimarus, and Condillac and Leroy operated, such as the difference between the French and German Enlightenment, and possibly many other historical factors. In this paper, I will not focus on sociological and historical factors influencing eighteenth-century naturalists but adopt a strong philosophical perspective on the eighteenth-century debate on animal cognition. I will argue that in order to fully understand the justification Buffon, Reimarus, Condillac, and Leroy gave for their respective positions, we must pay attention to the theoretical virtues these naturalists alluded to while justifying their position. These theoretical virtues have received little to no attention in the literature on eighteenth-century animal cognition, but figure prominently in the justification of the mechanist, instinctive, and sensationalist theories of animal behavior. Through my philosophical case study, we obtain a deeper philosophical understanding of the role of theoretical virtues in the eighteenth-century (life) sciences. I will show that theoretical virtues in the eighteenth century define what a good scientific theory looks like, and are in this respect similar to present day theoretical virtues, but were not always conceptualized as criteria for theory choice, even if they did in fact facilitate the choice of theories.

The topic of animal cognition in the eighteenth century is relatively little investigated. However, in recent decades a number of important studies have been published. Important work on Buffon has been written by Roger (1997a), who provides an impressive discussion of Buffon’s views on animals and the debate on the animal soul. Anita Guerrini (2012) has provided important insight into Buffon’s natural history of animals, examining the relationship between the works of Perrault, Daubenton, and Buffon. Robert J. Richards has given an account of controversies over animal instinct and intelligence in the seventeenth, eighteenth, and nineteenth centuries (Richards 1979, 1987). Richards classifies the controversy in the eighteenth century as a debate between Aristotelians, who strictly distinguished between the rational human soul and the animal soul guided by mere sensory cognition, Cartesians, who adhered to the doctrine of the animal machine, and sensationalists who allowed for animal intelligence. These positions correspond to the three positions I distinguish in this paper. Charles Wolfe (2017) has investigated the interactions between debates over the basic properties of matter, i.e., debates over various versions of materialism, and the blurring of the human/animal boundary, whereas Duchesneau (2017) investigates the relations between Haller’s identification of irritability and sensibility as distinct properties on the conception of animals and man. Gaukroger (2017) discusses the naturalization of sensibility in the eighteenth century, providing discussion of the works of Condillac, whereas Cheung (2017) discusses the work of Cabanis on reaction centers in living systems. Cheung (2006) provides a clear discussion of Reimarus’ theory of animal drives, Ingensiep (1996) provides an overview of the debate on animal souls in eighteenth-century Germany, and van den Berg (2018a) sketches the views on animal cognition of Buffon, Reimarus, and Kant, focusing, in contrast to this paper, mainly on the views on animal and human psychology of these authors. Finally, Riskin (2016) has written about the history of mechanism and agency in historical debates concerning animal cognition. A common thread of many of these papers is the focus on how conceptions of animal cognition influence our views on human cognition and the human/animal divide. I will, by contrast, focus mainly on the empirical and theoretical reasons that were given for justifying specific theories of animal cognition.

In this paper, I contribute to the existing research by adopting a philosophical perspective and by highlighting the role that theoretical virtues played in the justification of different scientific theories concerning animal cognition. As we shall see, theoretical virtues played an important role in the justification of the theoretical positions adopted by Buffon, Reimarus, Condillac, and Leroy. Although we cannot be certain that they played a role in the context of discovery, i.e., in the discovery of the different theories, they certainly played a role in the justification of these theories after they were formulated. Nevertheless, the theoretical virtues that informed the positions of these naturalists have not been studied. A full account of the theoretical reasons for espousing different theories on animal cognition in the eighteenth century thus needs to consider these theoretical virtues. This does not mean that Buffon, Reimarus, Condillac, and Leroy *only* appealed to theoretical virtues to justify their positions. As we shall see, they rejected the theories of opponents because they were based on false theoretical premises. Moreover, there are many sociological and historical factors that influenced the eighteenth-century debate on animal cognition, which I cannot discuss within the scope of this paper. However, theoretical virtues nevertheless played an important role in the justification of theories of animal cognition, as I will argue.

The project I am engaging in presupposes that eighteenth-century naturalists actually alluded to theoretical virtues when justifying their theoretical positions. This presupposition is not evidently true. Can we be sure that theoretical virtues of scientific theories as they are discussed in contemporary debates, i.e., virtues that good scientific theories must satisfy and that guide theory choice, are actually employed in the life sciences of the eighteenth century? Were theoretical virtues as we know them now accepted in the eighteenth century? In the present paper, I will argue that there is indeed solid historical evidence for claiming that eighteenth-century naturalists alluded to theoretical virtues when justifying their positions. I will provide an overview of contemporary theoretical virtues discussed in the philosophy of science and historicize these virtues in order to show that eighteenth-century authors had ideas about the virtues of a good scientific theory that are similar to our modern day ideas on the virtues that a good scientific theory must satisfy. However, I also show that theoretical virtues in the eighteenth century were not always conceptualized as criteria for theory choice, even if they did in fact function as such criteria. In this respect, the conceptualization of theoretical virtues in the eighteenth century differed from how theoretical virtues are conceptualized by contemporary philosophers of science. In this way, my paper will shed new light on the status and role of the theoretical virtues that eighteenth-century authors thought a good scientific theory must satisfy, contributing to knowledge of the history of theoretical virtues in science. After having identified and historicized the theoretical virtues, I will analyze the texts of Buffon, Reimarus, Condillac, and Leroy in order to verify the historical hypothesis that theoretical virtues played a role in the justification of eighteenth-century theories on animal cognition. My focus will be on the context of justification of eighteenth-century theories of animal cognition, and not on the context of discovery.

The structure of this paper is as follows. In Sect. 2, I analyze contemporary theoretical virtues in philosophy of science and I subsequently historicize them. In Sect. 3, I discuss Buffon’s views on animal cognition. I describe his mechanistic theory of animal behavior and consequently show which theoretical virtues justified Buffon’s mechanism. Section 4 discusses Reimarus’ theory of animal instinct and shows which theoretical virtues justified Reimarus’ theory. In Sect. 5 I discuss the views of Leroy and Condillac and show which theoretical virtues justified their attribution of reason or intelligence to animals. Finally, Sect. 6 provides a comparison of the different virtues that the naturalists refer to.

## Theoretical virtues then and now

In this paper it will be argued that theoretical virtues played an important role in the justification of different eighteenth-century theories on animal cognition. In this section, I first discuss contemporary theoretical virtues that play a role in my historical analysis. These theoretical virtues function as an interpretative model for studying the arguments of eighteenth-century naturalists. I will then historicize these theoretical virtues and demonstrate that eighteenth-century philosophers and scientists had methodological ideas about good scientific theories that are similar to the theoretical virtues that I use in my analysis. This shows that it is historically adequate to analyze eighteenth-century debates on animal cognition on the basis of the theoretical virtues that I have identified, while also providing new historical insight into the eighteenth-century philosophical views on the theoretical virtues that a good scientific theory must satisfy.

In order to identify the theoretical virtues that Buffon, Reimarus, Condillac, and Leroy use to justify their theories, I have carefully analyzed the arguments these naturalists offer for their positions in their texts and abstracted (from a modern interpretative perspective) the virtues that they explicitly or (often) implicitly refer to. Hence, historical investigation formed the basis for the identification of the theoretical virtues. For the sake of conceptual clarity, it will, however, also be useful to give definitions of the theoretical virtues I have identified. These definitions can be taken from the work of Keas (2018), who has provided a systematization of contemporary theoretical virtues. These theoretical virtues describe what a good scientific theory looks like and function as criteria of theory choice. The theoretical virtues that are important for this paper are the following: (i) *evidential accuracy*, which means that “a theory (T) first the empirical evidence well” (Keas 2018, p. 2762). (ii) *Causal adequacy*, which means that “T’s causal factors plausibly produce the effects (evidence) in need of explanation” (ibid.). (iii) *Simplicity*, which means that “T explains the *same facts* as rivals, but with *less* theoretical content” (ibid.).^1^ (iv) *Unification*, i.e., “T explains *more kinds of facts* than rivals with the *same* amount of theoretical content.” (ibid.). Another virtue that will figure prominently in this paper is (v) the virtue of *scientific understanding*, which has recently been analyzed by de Regt (2017). According to de Regt, there are two varieties of scientific understanding: (a) the understanding of nature or of a phenomenon (UP), which boils down to having an adequate explanation of the phenomenon. There is also (b) the understanding of a theory (UT), which boils down to being able to use a theory, e.g., by constructing models of a theory or by being able to perform deductions from theoretical principles (de Regt 2017, p. 23). According to de Regt, a scientist understands a theory if the theory is *intelligible* to her. Intelligibility can be defined as “the value that scientists attribute to the cluster of qualities of a theory (in one or more of its representations) that facilitate the use of the theory” (de Regt 2017, p. 40). As we shall see, Buffon, Reimarus, Condillac, and Leroy referred to several of these theoretical virtues in order to justify their theories.

Did these theoretical virtues also figure in eighteenth-century debates about what a good scientific theory must look like? In the following I will historicize the theoretical virtues and argue that this is indeed the case. I will discuss the works of the influential German philosopher and mathematician Christian Wolff (1679–1754), the methodological ideas on science of the French naturalist Buffon (1707–1788), the logical works of the German philosopher and logician Georg Friedrich Meier (1718–1777) and the German naturalist and logician Reimarus (1694–1768), the logic of Condillac (1714–1780), the philosophical works of Immanuel Kant (1724–1804), and the Encyclopedia of Diderot and d’Alembert.

Christian Wolff took proper sciences to be axiomatically structured systems of propositions with a clear distinction between fundamental and non-fundamental propositions (see for a model of this idea of science, De Jong and Betti 2010. See on Wolff’s axiomatic conception of science, Van den Berg and Demarest forthcoming, on which the account of Wolff given in this section is based). Wolff defines science as the “habit of demonstrating propositions, i.e., the habit of inferring conclusions by legitimate sequence from certain and immutable principles (Wolff [1728] 1963, p. 17). A demonstration is a deductive (typically syllogistic) inference from certain first principles (Wolff [1754] 1978, p. 172–173), and science thus consists in deducing propositions from fundamental and certain propositions. Reimarus, who typically follows Wolff’s views on science and logic, likewise construes proper science as axiomatic. According to Reimarus, in a proper science we start from universal axioms and demonstrate consequences through series of strict logical inferences (Reimarus 1766a, pp. 247, 345–346).

In physics, Wolff argues, fundamental propositions are discovered and established on the basis of experience and experiment (see for detailed discussion van den Berg and Demarest forthcoming). According to Wolff, in experimental physics we start from certain experiences or experiments and we then, employing the analytic method and reasoning from effects (consequences) to causes (principles), logically deduce the fundamental principles of physics. In this way, experimental physics establishes the principles of physics (Wolff [1728] 1963, pp. 53–54). Reimarus, again following Wolff, similarly takes physics to be based on observation and experiment and argues that the principles of physics are derived from experience, yielding so-called axioms of experience (Reimarus 1766a, pp. 226, 231, 233–234, 239). In what Wolff calls dogmatic physics, the empirical principles established in experimental physics function as axioms, from which we, employing the synthetic method and reasoning from the causes (principles) to the effects (consequences), deduce or demonstrate consequences. Importantly, Wolff states that the propositions that we demonstrate in physics must also be verified through experience. Through this procedure, we obtain what Wolff calls a *harmony between reason and experience* (De Angelis 2018, p. 340). In other words, when we prove a proposition in physics we should provide (a) a demonstrative proof of the proposition from first principles, and (b) verify the proposition by means of experience or experiment. Hence, experience and experiment play a twofold role in physics: they (i) provide the starting point on the basis of which we prove the principles of physics, and (ii) they verify propositions that are (syllogistically) demonstrated in physics. That experience is used to verify propositions of physics is also evident from Wolff’s account of hypotheses. According to Wolff, hypotheses gain in probability be logically deducing consequences from them and verifying them on the basis of experience (Vanzo 2015, p. 236). Reimarus adopted a very similar account of hypotheses (Reimarus 1766a, p. 334).

The ideas on the role of experience and experiment in physics that we have analyzed above are also articulated in eighteenth-century textbooks on physics, such as Johann Peter Eberhard’s (1727–1779) *Erste Gründe der Naturlehre* (1753) (see van den Berg and Demarest forthcoming). These methodological rules were not taken to only apply to physics, but they concerned natural science as a whole, including the life sciences, the latter being discussed by Wolff in his physics and by Eberhard in his textbook on physics (van den Berg 2014, pp. 66–69, 160–163) We can thus conclude that in natural science Wolff and the Wolffians, including Eberhard and Reimarus, adhered to methodological ideas that are similar to the theoretical virtue of evidential accuracy, which means that “a theory (T) fits the empirical evidence well” (Keas 2018, p. 2762).

Buffon also assigned experience and experiment an important role in natural science. Although it is generally agreed that Buffon did not accept an axiomatic ideal of science like Wolff (see Hoquet 2010), Buffon did try to establish a philosophical physics that is not merely descriptive but that elucidated the causes of natural phenomena (Hoquet 2010, pp. 45–48). In such a philosophical physics, Buffon claimed, there is no other master than experience (Hoquet 2010, p. 42). As Buffon puts the point in his “Premier Discours”, the true method of natural history involves the “complete description and exact history of each particular thing” (Buffon 1976, p. 157). However, natural history is not limited to exact descriptions of facts, as Hoquet has also stressed. As Buffon puts the point:

But we must try to raise ourselves to something greater and still more worthy of our efforts, namely the combination of observations, the generalization of facts, linking them together by the power of analogies, and the effort to arrive at a high degree of knowledge. From this level, we can judge that particular effects depend upon more general ones; we can compare nature with herself in her vast operations; and, finally, we are able to open new routes for the further perfection of the various branches of natural sciences. (Buffon 1976, pp. 171–172)

Hence, like Wolff, Buffon saw experience as the starting point of all natural science, on the basis of which we derive more fundamental or more general principles that elucidate the causes of natural phenomena.

The truths of the physical sciences, Buffon argues, are, unlike mathematical truths, based on facts of repeated observation and experiment (Buffon 1976, pp. 174–175). Hence, propositions of natural history must match experience and Buffon accepts ideas similar to the theoretical virtue of evidential accuracy. Buffon further notes that we can sometimes, in certain restricted domains of enquiry, infer the causes of phenomena through the union of physics with mathematics (Ibid., pp. 175–176). In physics, we can conjecture that a certain cause brings about a consequence. We can then apply mathematics to conjecture how often this effect happens in conjunction with its cause. If the conjecture “accords with the observations, the probability that you have guessed correctly is so increased that it becomes a certainty” (ibid., pp. 176). Through this procedure we can infer to the existence of a causal relation. This example again shows that Buffon thinks that propositions in natural science should match experience: it is the fact that our conjectures “accord with the observations” that allows us to establish certain propositions concerning causal relations in physics. Hence, like Wolff, Buffon adheres to methodological ideas that are similar to the theoretical virtue of evidential accuracy.

Condillac can also be said to accept ideas that are similar to the contemporary virtue of evidential accuracy. In his logic, Condillac notes that children have simple wants and test their judgments on the basis of observation, thus obtaining knowledge of nature (Condillac 1809, pp. 7–8). This is a natural logic that, according to Condillac, we should also follow in the sciences: “It will be sufficient to continue as nature made us begin, that is, to observe and to put our judgments to the test of observation and experience” (Condillac 1809, p. 10). The proper method of the natural sciences is thus to reason from experience and to verify propositions, such as hypotheses, by observation and experiment (ibid., pp. 31, 54, 129).

The contemporary theoretical virtue of causal adequacy states that the causal factors of a theory produce the effects in need of explanation (Keas 2018, p. 2762). Wolff and Buffon accepted the idea that in natural science we should provide *causal explanations* of phenomena. In his *Preliminary Discourse*, Wolff distinguishes between historical and philosophical knowledge (see for discussion van den Berg and Demarest, forthcoming). Historical knowledge provides bare knowledge of the fact, i.e., knowledge that shows *that* something is the case. Philosophical knowledge, which is the type of knowledge we strive for in the sciences, provides the reason or ground for the fact and explains *why* the fact obtains (Wolff [1728] 1963, pp. 4–5). In physics, which is a part of philosophy, we must specify the causes of effects, explaining why they obtain. For example, Wolff states that Newton showed that gravity is the cause of the elliptical motion of the primary planets, providing the reason for why primary planets describe elliptical orbits (Ibid, p. 5). As we have shown above, Buffon also wanted to establish a physics that specifies the causes of phenomena (Hoquet 2010, pp. 45–48). Hence, both Wolff and Buffon accepted something similar to the theoretical virtue of causal adequacy.

Something akin to the theoretical virtue of simplicity, a principle of economy, was also accepted in eighteenth-century philosophy and science. In the encyclopedia of Diderot and D’alembert, in the lemma on system, it is said that the principles of a systematic science must be few in number:

A *system* is nothing more than the arrangement of the different elements of an art or a science in an order that makes them mutually dependent; the primary elements lead to and account for the final ones. Those which explain the others are called *principles*, and the *system* is all the more perfect as the principles are fewer in number: it is even to be desired that they be reduced to one. For just as there is a principal spring in a clock on which all the others depend, there is also in all *systems* a first principle to which the different elements that compose them are subordinated. (“System” 2009 [1765])

A similar principle of economy was accepted by the German philosopher immanuel Kant, who argued that science is guided by the principle of genera or homogeneity, according to which species should be subsumed under genera until we reach a highest genus (KrV A 651-2/B679-80). This maxim prescribes us to base sciences on a minimum number of principles.

In his *Vernunftlehre* (1752), the German philosopher and logician Georg Friedrich Meier discussed several perfections of scientific knowledge (These perfections, and the position of Kant as described in this paper, are extensively discussed in van den Berg’s unpublished “Kant’s Ideal of Systematicity in Historical context”, on which the following account is based. On Kant’s construal of these perfections, see also Falkenburg 2000). These perfections were ideals of scientific knowledge, and the more a science satisfied these perfections the more perfect it was. One perfection was the perfection of fruitfulness. Meier argued that if a proposition or principle has many consequences, it is fruitful (Meier 1752, 101). In science, we should, according to Meier, search for propositions and principles that allow for the derivation of the maximal number of consequences. Combined with the principle of parsimony or simplicity, we thus obtain the maxim that in science we should find the smallest set of principles that allow us to infer the maximum number of consequences. This combined maxim was once again adopted by the philosopher Kant, who argued that in science we should balance the regulative maximin of genera or homogeneity, which, as we have seen, directs us to search for a minimum of principles, and the regulative maxim of species or specification, which, when applied to propositions, directs us to infer a maximum number of consequences (KrV A654-55/B682-3). The idea that we should derive a maximum of consequences on the basis of a minimum of principles, as adopted by Meier and Kant, is akin to the modern idea of unification, which tells us that we should explain a maximum of facts in terms of a limited number of principles (Keas 2018, p. 2762). In this way, Newton, for example, gave a unified explanation of planetary motions, the tides, the motion of comets, etc. in terms of the principle of gravity and the laws of motion.

Finally, we can turn to the theoretical virtue of scientific understanding. Wolff pays special attention to understanding, noting that in science we obtain understanding (*verstehen*) of phenomena if we know the grounds of these phenomena, i.e., the reason for *why* these phenomena obtain (Wolff [1751] 2003, pp. 105–106). This means that in natural science scientific understanding is the result of providing causal explanations. If we know, through Newton, that gravity is the cause for why planets describe elliptical orbits, we automatically obtain scientific understanding of the orbits of planets. Hence, Wolff adopted something akin to what de Regt calls the understanding of nature or of a phenomenon (UP), which follows from having an adequate explanation of the phenomenon. I have not found explicit eighteenth-century theoretical discussion of what de Regt calls *intelligibility* or the understanding of a theory (UT), which means that scientist are able to use a theory, e.g., by constructing models of a theory (de Regt 2017, p. 23). Nevertheless, we shall see that naturalists in the eighteenth century alluded to intelligibility or the understanding of a theory (UT) in order to justify their positions.

How were these theoretical virtues used in the eighteenth century and what was their status? In order to answer this question, we can have a look at Meier’s discussion of the logical perfections of knowledge, which resemble our present day theoretical virtues. Meier discusses these perfections in his logic and notes that the purpose of logic is to teach scholars how to think philosophically, logically (*Vernünftig*), and learned (*gelehrt*) (Meier 1752, Vorrede). He argues that humans, having reason, have a special place in the world and that in accordance with this special place humans should seek to perfect knowledge of the world as much as possible (Ibid.). The goal of logic, and of the logical perfections discussed in logic, such as simplicity and fruitfulness, is thus to perfect knowledge as much as possible in order to obtain adequate and perfect knowledge of the world (Ibid., pp. 3–5).

According to Meier, scientific knowledge can possess different logical perfections in different degrees, and the goal of scholars is to obtain proper scientific and philosophical knowledge, which possesses these perfections in a very high degree (ibid., p. 6). Hence, the logical perfections describe an ideal of scientific knowledge and prescribe rules that define what proper science or proper philosophical knowledge is (ibid.). As such, the logical perfections resemble our present day theoretical virtues, which describe what a good scientific theory is. There are, however, also differences between the philosophical views of eighteenth-century authors on theoretical virtues or logical perfections and our modern understanding of theoretical virtues. The most salient difference is that, whereas contemporary philosophers view theoretical virtues as criteria of theory choice, I have found no explicit statement in the eighteenth century that theoretical virtues or logical perfections allow us to decide between different theories. In principle, the theoretical virtues or logical perfections of the eighteenth century could also function as criteria of theory choice, and in the following sections we will see that they do. However, in the works of Meier there simply is no explicit discussion of the virtues of good scientific theories that indicate that they function as criteria for theory choice. Where Meier’s discussion also differs from our present day conception of theoretical virtues is that Meier, like other eighteenth century authors, does not endorse the idea of underdetermination of different scientific theories by the empirical data, and that, given this underdetermination, we can appeal to theoretical virtues in order to facilitate theory choice. In spite of these differences, the logical perfections do define what counts as a good scientific theory, and this fact suggests that scientists in the eighteenth century who knew of these perfections would appeal to the logical perfections or theoretical virtues in order to justify their scientific theories. In the next sections, we will provide evidence for this historical hypothesis by studying eighteenth-century debates on animal cognition.

## Buffon’s mechanism

In recent years, Buffon has sometimes been presented as a critic of mechanism (Reill 2005, p. 38–40). However, as we shall see below, in the “Dissertation on Animals” and “Homo Duplex”, Buffon clearly tried to give a mechanical account of animal behavior (see also Roger 1997a, p. 240). This is clearly illustrated by many passages, including the following, where Buffon argues that the uniformity of animal behavior proves that animals are governed by mechanism:

Whence proceeds this uniformity in all the operations of animals? Why does every species perform the same work in the very same manner? And why is the execution of different individuals neither better nor worse than that of every other? Can there be a stronger proof that their operations are only the result of pure mechanical impulse? (Buffon 1785a, p. 365)

Buffon’s mechanism is illustrated by his account of the behavior of higher animals.^2^ Buffon attempted to explain this behavior by providing a mechanical account of animal movement that was modeled on Newton’s third law of motion. According to this picture, objects act on animals by means of the senses, and animals react by means of motion (Buffon 1785b, p. 218, p. 220). More specifically, the action of external objects on the sensory organs results in the animal having sensible impressions. These impressions are momentary vibrations in the sensory organs, i.e., vibrations that exist for a short period of time. The impressions (vibrations) are transmitted to the brain (the internal sense).^3^ In the brain, the impressions produce distinct and durable vibrations (ibid., p. 232). The brain thus retain*s* different impressions (vibrations) for a relatively long time (ibid., p. 225). The brain also integrates different impressions and guides the animal to react to external circumstances. Importantly, the impressions in the brain give rise to appetite (desire) or disgust (aversion), depending on the internal state of the animal. Appetite leads an animal to move toward an object, whereas disgust leads to animal to flee from an object. When an animal has been determined by appetite or disgust, the brain transmits its vibrations to the nerves, which guide the muscles and the motion of the animal (ibid, p. 232).

On Buffon’s account, then, *impressions*, *the brain* (internal sense), and *desire and disgust* are the causes of animal behavior. Buffon ultimately claimed that the brain or internal sense is sufficient to explain all the actions of animals:

From these facts it appears, that, in brutes, the internal sense differs only from the external senses, by the faculty it possesses of retaining received impressions. This faculty is alone sufficient to explain all the actions of animals, and to give us some idea of what passes within them. (Buffon 1785b, p. 229)

The question was how, if we adopt this simple mechanical picture, we can explain that animals exhibit behavior that is similar to human behavior, which is guided by intellect.

To explain why animal behavior is similar to human behavior, Buffon argued that animals learn by *association* and *imitation*. Consider a dog which, though “excited by the most violent appetite” (ibid., p. 235), refuses to wrest food from the hand of his master and starts to frisk. This phenomenon provides a problem for Buffon’s theory: the dog’s desire should lead him to seize the food. The fact that the dog does not do so may indicate that it reasons. According to Buffon, however, the dog has learned by association, i.e., instrumental conditioning, that if it obeys its desire it will suffer blows. The dog’s earlier attempts to take the food have been uniformly accompanied by “impressions of pain”, and this association is retained in the internal sense (ibid, p. 237). Consequently, the animal does not act but “remains in equilibrio” (ibid). However, the dog has also learned, again by instrumental conditioning, that if he frisks his master will give him the food. These associations explain the dog’s behavior. Since association can be mechanically understood in terms of the action of the inner sense, Buffon thinks that the internal sense can “produce every motion they perform” (Buffon 1785b, p. 238).

Next to associative learning, Buffon ascribed learning by *imitation* to animals. Most animals only imitate animals of the same species. This explains, for example, how birds learn to fly. Some higher animals, apes for examples, are capable of imitating other animals, such as humans. The capacity for imitation can again be mechanically explained in terms of the action of the internal sense. Buffon claims that since humans and animals with the most delicate senses are the best imitators, imitation “depends on the vivacity with which the internal material sense receives the impressions of objects” (ibid, p. 281). Moreover, since human children are good at imitation without employing reflection, the fact that animals can imitate does not imply that animals have the capacity for thought or reflection.

### Buffon’s justification of mechanism

Why does Buffon value mechanism? In the following, I will argue that Buffon accepted mechanistic accounts of animal behavior because of metaphysical reasons, moral reasons, and a particular interpretation of empirical data concerning animal behavior. In addition, I show that Buffon justified his account through an appeal to ideals of explanation and different theoretical virtues that support his mechanist explanation of animal behavior. I will start by explaining the empirical data that support Buffon’s mechanist theory.

Buffon argued that animal motion and behavior is relatively uniform. Presented with the same stimulus (e.g., food), animals will normally react in the same manner (e.g., moving toward the food). The lack of variability in animal behavior is a reason for (i) claiming that there is no strong analogy between the behavior of humans, who use reason to react differently to the same stimuli, and animals, and (ii) attributing a mechanism that is responsible for the animal behavior. As Buffon puts the point:

Animals never invent, nor bring any thing to perfection; they uniformly do the same things in the same manner. This destroys the force of the analogy [between man and animal] so much, that we may even doubt of its reality: We ought, therefore, to inquire, whether the actions of brutes proceed not from principles entirely different from those which actuate men. (Buffon 1785b, p. 236)

Buffon takes the uniformity of animal behavior to necessitate a mechanical explanation:

The certainty with which animals are supposed to act, and the stability and uniformity of their determinations, sufficiently evince them to be the effects of pure mechanism. To doubt, to deliberate, to compare, are the essential characters of reason. But movements and actions which are always decisive, and always certain, indicate, at the same time, both mechanism and stupidity. (Buffon 1785b, pp. 294–295)

Although Buffon took animal behavior to be relatively uniform, he did allow for some variability of animal behavior. Buffon acknowledged that animals learn through experience, which leads to variable behavior. However, although animals learn through experience, Buffon did not think that learning must be explained by attributing reason to animals. The reason is, as we have seen in the previous section, that animal learning can be explained mechanically. Animals learn to associate certain sensations through instrumental conditioning, which can be explained purely in terms of the functioning of the internal sense (the brain). Thus, mechanism allows one to explain both the uniformity and the variability of animal behavior. Buffon thus thought that mechanism fits the empirical data of animal behavior well, because mechanism can explain all kinds of animal behavior and the limitations of animal behavior are explained by the fact that they result from mere mechanism. We can thus say that he appealed to the theoretical virtue of *evidential accuracy* to support his theory (Keas 2018). The question is why we should explain the variable behavior of animals in terms of mechanism rather than by attributing reason to animals: the fact that variable animal behavior can be explained mechanically does not imply that it should be explained mechanically. One of the reasons why Buffon insisted on a mechanist theory is moral and religious. He took the ascription of reason to animals to degrade humans, who he thought of as naturally superior to animals. Buffon expresses the superiority of humans as follows:

Man holds a legitimate dominion over the brute animals, which no revolution can destroy. It is the dominion of mind over matter; a right of Nature founded upon unalterable laws, a gift of the Almighty, by which man is enabled at all times to perceive the dignity of his being. (Buffon 1785b, p. 302)

In the following, however, we will see that apart from empirical and moral reasons, Buffon appealed to different ideals of explanation and different theoretical virtues to argue for mechanism.

### Buffon on mechanism and theoretical virtues

Buffon’s views on mechanical explanation can be distilled from his account of nutrition, growth, and reproduction, given in the second volume of the *Histoire naturelle*. Here, reproduction, as explained by the *Encyclopedia Brittanica* (1768), should be understood as the act whereby a thing is produced anew, or grows a second time, and thus includes forms of regeneration of organic parts.^4^ Buffon explains nutrition, growth, and reproduction on the basis of the action of *organic molecules*, *internal molds*, and (attractive) *penetrating forces*.^5^ Buffon’s explanation, as he stresses himself, was not mechanistic in the traditional Cartesian sense, since he argued for the existence of (Newtonian) penetrating forces (Buffon 1785a, p. 48). He also rejects the mechanistic maxim that in order to understand organic structure we must reduce it to “simple and unorganic parts” (Ibid, p. 19). Still, Buffon himself took his account of nutrition, growth, and reproduction to be an extension of the traditional mechanical philosophy (ibid, pp. 47–48).

Buffon’s explanation of nutrition, growth, and reproduction is instructive because it sheds light on Buffon’s views on the virtues that a scientific explanation must satisfy. First, Buffon stresses that his explanation allows us to provide an account of the *general phenomenon* of reproduction, i.e., it is applicable to the reproduction of different living beings. As Buffon puts the point with respect to the problem of reproduction:

And, without limiting our research to the generation of man, or of any particular animal, let us contemplate the general phaenomena of reproduction; let us collect facts, and enumerate the various methods employed by Nature for the renovation and transmission of organized existences. (Buffon 1785a, p. 16)

The fact that Buffon wanted to provide an account of reproduction that is applicable to different species highlights that he wanted to give a *unified* explanation of reproduction. Hence, Buffon appealed to the virtue of unification in order to support his mechanistic account of nutrition, growth, and reproduction.

Second, Buffon also stressed that he wanted to provide a *causal* explanation of nutrition, growth, and reproduction. As Buffon states:

[…] we are still under the necessity of asking, by what secret cause she [nature] enables beings to propagate their kinds? […] if we can conceive a method of reproduction, depending on primary causes, or which, at least, is not repugnant to them, we ought to be satisfied with it; and the more relation it has to the other effects of Nature, it will rest upon a firmer basis. By the nature of the question, then, we are permitted to form hypotheses, and to choose that which appears to have the greatest analogy to the other pheanomena of nature. (Buffon 1785a, pp. 29–30)

Hence, in explaining nature we must provide *causal explanations*. This can be taken to refer to the virtue of causal adequacy: a theory must provide causal factors that plausibly produce the effects in need of explanation (Keas 2018, p. 2762). Moreover, Buffon’s stress on analogies points once again to the fact that he valued *unified* explanations. According to Buffon, as shown in van den Berg (2018b), it is through discovering analogies between different phenomena, which lead us to provide a similar explanation for these phenomena, that we are able to provide unified explanations of these phenomena. Analogy is a means that allows us to discover general causes of different effects (Buffon 1785a, p. 28).

Third, Buffon takes his causal account of reproduction to provide epistemic insight into *how* animals and plants reproduce. Here, he thus refers to the epistemological advantages of providing causal explanations.

[…] if it be asked *how* animals and vegetables are reproduced? We are enabled to solve the question, by giving the history of the generation of every species of animal, and of the reproduction of every species of plant. (Buffon 1785a, p. 29)

Although Buffon does not state this explicitly, we may assume that he took mechanical explanations as adequate examples of explanations of how a phenomenon comes about, since mechanical explanations provide causal mechanisms that specify the processes of how something happens.

Fourth, Buffon’s account does not appeal to fictitious final causes. Appeals to final causes mistake the effect for the cause and hence must be rejected. We must provide explanations in terms of efficient causes. In short, Buffon rejected the metaphysics of final causes and hence preferred mechanistic explanations:

We must likewise reject every hypothesis which is founded on final causes, such as, that reproduction is ordained in order to replace the living for the dead […] for such hypotheses, in place of explaining the effect by physical causes, stand on no other foundation than arbitrary relations and moral affinities. (Buffon 1785a, p. 30)

Finally, Buffon valued his explanation of nutrition, growth, and reproduction because it allowed for *simple* and (again) *unified* explanations of different phenomena: nourishment, growth, and reproduction are shown to be “effects of the same cause” (Buffon 1785a, p. 44). Here, Buffon seems to appeal to what philosophers now call syntactic simplicity (Baker 2016): it is a virtue of a theory if it explains multiple phenomena in terms of a limited number of principles. This is done by explaining nourishment, growth, and nutrition in terms of a single cause. Moreover, by explaining these different phenomena in terms of a single cause, we also give a unified explanation of these different phenomena. Hence, considerations concerning simplicity and (once again) unification lead Buffon to praise his mechanist account of nutrition, growth, and reproduction.

Buffon’s mechanist explanation of animal behavior has the same characteristics as his account of nutrition, growth, and reproduction. Hence, I think we can understand Buffon’s preference for mechanical explanations of animal behavior by citing the previously established virtues of mechanical explanation. The search for mechanical explanations of animal behavior indicates that Buffon wished to explain *how* animal behavior occurs. Mechanisms, such as the explanation of animal behavior in terms of external stimuli and the action of the internal sense (see previous section), specify mechanical processes that elucidate how certain phenomena come about. Moreover, through appealing to mechanisms, we do not appeal to fictitious *final causes*. Buffon’s mechanical account of animal behavior is also a *causal* account of animal behavior that reflects the order of nature. Finally, Buffon’s mechanical account of animal behavior provided a *simple* and *unified* explanation. This follows from his insistence that all animal movements can be explained in terms of a single cause, namely the internal sense. As Buffon puts it: “this faculty [the internal sense] is alone sufficient to explain all the actions of animals” (Buffon 1785b, p. 229). In line with this prescription, Buffon also claims that we should seek to reduce animal behavior to a few causes. He claims that we should not attribute a multitude of distinct psychological causes resembling man to animals, but explain the actions in a more parsimonious manner in terms of a few (mechanical) causes. Such a *simple* explanation does justice to the intentions of the creator, who created animals in the most parsimonious way:

But, as the laws of Nature are general effects only, and, as the facts in question are limited and particular, it would be less philosophic, and more unworthy, of the ideas we ought to entertain of the Creator, to embarrass his will thus gratuitously with a vast number of petty statutes, of which one must be enacted for bees, another for owls, a third for field-mice &c. Should we not, on the contrary, exert all our efforts to reduce these particular effects to more general ones? (Buffon 1785b, p. 295)

Here, we see that Buffon argued that God created nature on the basis of few principles, and that this justifies our search for simple explanations of nature. As we shall see in the next sections, Reimarus adopted a similar justification of searching for simplicity in science.

In what precise sense did Buffon’s account of animal behavior support unification? We have already seen that Buffon’s mechanism provided an account of both the uniformity and variability of animal behavior. By doing this, Buffon’s mechanism provided a unified account of animal behavior. This appeal to unification is also apparent in Buffon’s claim that all animal behavior can be explained in terms of the internal sense (the brain). In addition, Buffon aims, I claim, to provide a unified account of animal behavior in the sense that the theory applies to all forms of animal behavior including *human* behavior. Recall that Buffon aims to show that animal behavior can be conceptualized as a reaction to impressions on our senses. He supports this claim by analyzing human behavior from a physical point of view, i.e., human behavior not subject to the influence of our rational soul. If we adopt this perspective, Buffon claims, “the movements we [humans] perform in consequence of desire, and the desire itself, proceed entirely from the impression made by the object upon our senses” (ibid, p. 221). As evidence, Buffon cites, among others, humans deeply occupied with study who unconsciously eat when their hand accidentally feels some bread. Hence, Buffon strives for mechanical explanations of animal behavior because it allows him to explain commonalities between the behavior of animals and humans, even though he is always quick to remark that humans have a rational soul (ibid, p. 224). He aims to provide mechanisms that underlie *all* forms of animal behavior, in order to determine both difference and similarities with other animals (ibid, p. 209). The upshot of this discussion is that Buffon thought that at least part of human behavior can be explained by the same mechanisms that explain animal behavior.

To sum up: Buffon justified mechanical explanations of animal behavior by referring to (i) the fact that mechanical explanations eschew final causes (metaphysical assumption), (ii) the fact that attributing intelligence to animals degrades humans (moral reason), (iii) empirical observations of the uniformity of animal behavior that fit a mechanistic explanation of animal behavior (the theoretical virtue of evidential accuracy), (iv) the fact that mechanisms show *how* animal behavior is possible (an ideal of explanation), (v) the fact that mechanisms provide a causal account of animal behavior reflecting the order of nature (the theoretical virtue of causal adequacy), (vi) the fact that the mechanical explanation of animal behavior is simple or parsimonious (the theoretical virtue of simplicity) (vii) the fact that the mechanical explanation of animal behavior unifies a range of different phenomena (the theoretical virtue of unification). It must be stressed that Buffon mainly refers to theoretical virtues for justifying his accounts after he established the accounts. Hence, they have a place within the context of justification and allow for the public justification and communication of his accounts. It is unclear whether they played an important role in the discovery and establishment of his theory.

### Buffon on simplicity and scientific understanding

I have argued that Buffon appeals to the theoretical virtue of simplicity to justify his mechanism. However, Buffon did not always seem to endorse simplicity as a theoretical virtue. This becomes clear in the “Premier Discours” (1749), a text famous for Buffon’s attack on Linnean systematics (See Sloan 1976, to which the following account is indebted). In this text, Buffon rejects taxonomies using a single feature of plants as a distinguishing characteristic (Buffon 1976, p. 151–152). Here one may think, for example, of Linnaeus’ method of logically dividing the plant realm into classes according to the number of *stamens* of plants (Müller-Wille 2007). This method yields defective classifications, because there are plants that do not have stamens and plants in which the number of stamens varies (Buffon 1976, p. 154).

Buffon also argues, employing a principle of plenitude (Lovejoy 1936), that it is *impossible* to adequately classify nature. If we study nature, we find that “it is possible to descend by almost imperceptible degrees from the most perfect of creatures to the most formless matter” (Buffon 1976, p. 150). Since nature is a continuum (Sloan 1976, p. 359), and one will always find many intermediate species, classifications are necessarily defective. On this basis, Buffon rejects, for example, Linnaeus’ division of the animal kingdom into six classes, noting that the more one *augments* the number of divisions, the more one approaches truth, since “in nature only individuals exist” (Buffon 1976, p. 164). Here, Buffon thus rejects ontological simplicity and simplicity of theory.

Although Buffon rejected simplicity in the “Premier Discours”, there is plenty of evidence that he accepted simplicity as a theoretical virtue in his account of animal behavior. Maybe Buffon changed his mind. It can be remarked that, as we have seen, Buffon strongly valued unification as a theoretical virtue. However, unification and simplicity are related (Sober 1988): by explaining multiple phenomena in terms of a few principles (simplicity) we also provide a unified explanation of these phenomena. Hence, it is difficult for Buffon to accept unification without also adopting simplicity as a theoretical virtue.

The “Premier Discours” provides more information about the theoretical virtues that Buffon adopts: it illustrates that Buffon adopted the theoretical virtue of scientific understanding, in the sense of understanding of nature (UP) and understanding of theory (UT). This becomes clear if we consider Buffon’s remarks about the proper method of natural history. Buffon argues that since existing taxonomies impose arbitrary relations to a complex world, we must develop a new method for natural history, where, instead of dividing nature on the basis of a *single* characteristic, we provide a “complete description and exact history” of objects (Buffon 1976, p. 157). When *describing* animals, we must completely describe *multiple* characteristics (ibid, p. 160). After the descriptive part, we provide a *history* of the species, including their conception, education, and so forth (ibid). By adopting this method, Buffon’s goal was to draw nearer to articulating a genuine natural system (Sloan 1976, p. 370).^6^

Finally, Buffon thinks that in natural history *man* serves as a model (Buffon 1976, p. 169). Praising Aristotle, he claims that we must base the order of our descriptions on objects “which interest us the most owing to the connections which they have with us” (Ibid, p. 162). Thus, in providing a natural history of animals, we start with animals *useful* to us, such as the horse, and then proceed to less familiar animals (Ibid, p. 161–162). This method is “easier, more agreeable, and more useful” than other methods (Ibid, p. 162). Here Buffon stresses the pragmatic advantages of his method. He also suggests that his method is superior to other methods because it better facilitates scientific understanding. His method is the “most natural of all”: it orders objects relative to the investigators “acquaintance with them, because that is indeed the order according to which he experienced them” (Buffon 1976, p. 162). It is in this context that Buffon remarks that his method is a “simple and natural manner of considering things” that is “preferable to more recondite and complex methods”, such as those of Linnaeus (Ibid, p. 162). Hence, theories and methods that are *suited to the investigator*, *easy* and *simple for the investigator*, *agreeable*, and *useful*, are to be preferred to more complex theories. Here, I think, Buffon refers to two types of scientific understanding. He thinks that the *theory* must be understood by the investigator (UT) insofar as the investigator must be able to easily use the theory. This is expressed by saying that the theory must be suited to the investigator and simple for the investigator. In addition, Buffon thinks that his method better describes the order of nature than the method of Linnaeus and thus thinks that his theory provides scientific understanding of the *objects of nature* (UP): it provides the best classification or explanation of the order of nature. Buffon thus definitely considered scientific understanding in these two senses to be theoretical virtues. What type of scientific understanding does mechanism provide? We can be sure that Buffon took mechanism to provide scientific understanding of the objects of nature (UP, in this case: animal behavior) since these objects are properly explained by mechanism. Buffon does not, however, explicitly state that mechanism provides an investigator with scientific understanding of a theory (UT), since he does not claim that mechanistic theory is somehow easier to use or to understand than alternative theories (e.g., mentalistic theories).

To conclude, Buffon appealed to empirical data, moral reasons, metaphysical assumptions and theoretical virtues to support his mechanism. The following theoretical virtues were taken to support his mechanism: (i) evidential accuracy, (ii) causal adequacy, (iii) simplicity, (iv) unification, and (v) scientific understanding of nature (UP). In the next two sections, we will describe the reasons and theoretical virtues for adopting explanations of animal behavior in terms of instincts and in terms of reason.

## Reimarus on animal cognition

In the previous section we have seen how Buffon developed a mechanist perspective on animal cognition. In this section, we will discuss how Buffon’s analysis of animals was taken up by Hermann Samuel Reimarus, who wrote one of the more influential works on animal instinct in the eighteenth century.

Hermann Samuel Reimarus was born in Hamburg on 22 December 1694, and studied theology, philosophy, and ancient languages in Jena (van den Berg 2013, van den Berg 2018a, Lötzsch 1979). In 1728, he became Professor of Hebrew and Oriental Languages at the Gymnasium in Hamburg (ibid.). Reimarus was an influential scholar. In 1754, he published a work on natural religion, *Die vornehmsten Wahrheiten der natürlichen Religion* (1754), followed by a book on logic, the *Vernunftlehre* (1756). His *Allgemeine Betrachtungen über die Triebe der Thiere*, a work on animal instinct, was published in 1760 (van den Berg 2013, Van den Berg 2018a, Lötzsch 1979).

Reimarus explained all animal behavior in terms of animal drives or instincts (Reimarus 1762, pp. 2–3; cf. Jaynes and Woodward 1974a, b; Cheung 2006, Zammito 2018, pp. 134–144. Van den Berg 2018a). Drives were taken to govern the efforts of animals toward specific actions. Reimarus’ claim that all animal behavior must be explained by innate drives or instincts was predicated on the idea that animal behavior cannot be explained by mechanism alone. In the next sections, we will consider Reimarus’ theological background and his critique of (Buffon’s) mechanism, and discuss some of the reasons why Reimarus adopted his theory of instinct. A full account of the reasons for accepting the theory of instinct, including the theoretical virtues which Reimarus employs to justify his theory, will be given after this.

### Reimarus’ theological background

Reimarus’ work on animal drives or instincts was strongly influenced by his theological beliefs. These beliefs were expounded most clearly in his *Die vornehmsten Wahrheiten der natülichen Religion* (1754), and have recently been discussed clearly by Zammito (2018). The aim of this work, as Reimarus explained in the preface, was to counter the predominantly French materialism that attacked Christianity and morality (Reimarus 1766b, p. 3; Zammito 2018, p. 134). Among the thinkers that Reimarus heavily criticized were Buffon and De La Mettrie. Against atheist materialism, Reimarus insisted that God created the world, that God created living organisms, and that nature was thoroughly teleologically ordered. Reimarus specified several axioms (*Grundsätze*) of natural religion that aptly summarize his beliefs (Reimarus 1766b, pp. 301–302). These axioms included the following: (i) lifeless objects exist for the sake of the living, (ii) a body exists for the sake of its soul, (iii) everything that happens in the world exists in accordance with an intention of God, etc. These axioms show that Reimarus thought of nature as teleologically ordered in accordance with divine intentions. Accordingly, if we want to understand nature, we must understand the teleological structure of the world.

A common feature of Reimarus’ critique of materialism was his insistence that purely materialistic and mechanistic explanations cannot account for the observed teleological order in nature. Thus, for example, he argued that pure chance cannot give rise to a purposefully organized organism, just as a random ordering of letters cannot give rise to the Aeneid (Reimarus 1766b, pp. 115–116). Moreover, he argued that animals cannot be machines and must have a soul since mechanism cannot explain the purposeful behavior of organism (Reimarus 1766b, p. 331). He took the complex purposive organization of organisms and the complex purposive behavior of animals as sure signs of divine creation and order (Reimarus 1766b, pp. 310–311). Indeed, Reimarus thought that the idea that animal behavior was governed by preformed and innate animal instincts provided proof of divine creation: it was God who created animal instincts and made sure that the behavior governed by these instincts was perfectly adapted to the environment. Given Reimarus insistence on the teleological order of nature, it is also not surprising that against Buffon he insisted that it was a mistake not to appeal to final causes in science (Zammito 2018, p. 136).

In short, Reimarus theological beliefs strongly influence his views on animal instincts and his opposition to figures such as Buffon and De La Mettrie. In the next section, we will take a closer look at Reimarus’ views on the mechanical inexplicability of animal behavior, which is one of the main reasons for arguing that animal behavior must be explained by reference to instinct. After that, we will consider the theoretical virtues that influence Reimarus’ theorizing.

### Reimarus on mechanism and instinct

In his *Critique of Judgment* (1790), Immanuel Kant famously claimed that organisms do not allow of mechanical explanation. Kant’s predecessor Reimarus likewise limited the scope of mechanical explanations in biology and psychology.^7^ Reimarus’ anti-mechanism sets him apart from thinkers such as Buffon even if he agreed with Buffon that animal behavior should be explained in terms of low-level psychological processes.

To understand Reimarus’ position, we may first note that he explicitly attacked Buffon’s mechanism in his *Religion*. In this work Reimarus argued that instincts are innate and hereditary capacities of animals to perform behavior that maintains the individual and preserves the species. Moreover, instincts are divine gifts. Reimarus’ criticized Buffon for eliminating God from natural history and for explaining all animal actions in terms of the vibrations of the brain (Reimarus 1772, pp. 346–360). Against Buffon, Reimarus simply posited that psychological capacities cannot be mechanically explained. In addition, Reimarus’ argued that Buffon’s mechanism, which explains animal behavior in terms of *desires* and *aversions*, cannot explain how animals have “a disposition to the most proper conduct to obtain” certain goods.^8^ Reimarus thus rejects mechanist accounts of instinctive behavior, provided by the likes of Buffon and, we may add, by de la Mettrie, who in his *Histoire Naturelle de l’ame* (1745) defined instinct as consisting in *purely mechanical* bodily dispositions (De la Mettrie 1745, p. 143).

The claim that psychological faculties and capacities are mechanically inexplicable is made explicit in Reimarus’ *Triebe*. There, Reimarus notes that when the sensory organs of humans are affected by sensible impressions, a natural endeavor of the *soul* causes us to have *representations*, i.e., to have an image (*Bild*) of objects. The soul must be responsible for having representations since we are *conscious* of what is represented, and consciousness, Reimarus claims, is something that is wholly inexplicable in terms of mechanical rules (Reimarus 1762, 26). Mechanism thus does not suffice even at the basic level of explaining how we (and other animals) can have representations. The upshot of this argument is that other psychological faculties, e.g., the capacity to *associate* representations, to *memorize* representations, etc., cannot be mechanically explained.

Reimarus also took different *adaptations* to be mechanically inexplicable. He argued that both the complex *structure* and the instinctive *behavior* resist mechanical explanation. According to Reimarus, the parts of animals are perfectly adapted to each other, to the external environment, and to the mode of life of animals. Thus, for example, birds that prey on fish must have wings, a long neck, and feet that allow them to swim. This adaptedness of (the parts of) organisms was incomprehensible on blind natural laws alone, and provided a sure sign of God’s wisdom and design (Reimarus 1762, pp. 11–12). Reimarus also argued that the complex *behavior patterns* of animals were perfectly adapted to their mode of life. Each animal has a particular way of living, i.e., it lives in its proper element (water, earth, etc.), it has its own habitat, its particular way of nest building, and so forth. The behavior of animals must be adapted to this way of living, e.g., to their element, habitat, and so forth, or animals will not be able to preserve their own species. For this reason, Reimarus concluded that the behavior of animals is *imperfectable* (ibid., pp. 124–125). If the behavioral repertoire of any animal would not be perfectly adapted to the animal’s mode of life, then no animal would survive. The perfect adaptedness of animal behavior again provided evidence of God’s wisdom and design, and could not be explained by appeal to mechanism alone.

To substantiate the claim that animal behavior is mechanically inexplicable, Reimarus rejected Buffon’s mechanist attempt to explain all animal actions in terms of desire (appetite) or disgust (aversion). According to Reimarus, and here he agreed with Buffon, all animals are governed by a fundamental drive called self-love. Any animal will represent objects that provide sensible pleasure (*Lust*) as good and will be inclined to seize this object. Alternatively, objects that arouse pain will trigger avoidance behavior. The approach to objects that give pleasure and avoidance of pain is necessary for the preservation of the animal and its species (ibid., pp. 60–62).

In contrast to Buffon, however, Reimarus did not believe that animal behavior can be fully explained in terms of pleasure and pain that arise through external impressions. Reimarus invoked many instances of animal behavior that appears to be uncaused by external stimuli to make this argument (we might call such behavior, following Allen and Bekoff (1997), relatively *stimulus*-*free* behavior). For example, egg-lying birds care for their eggs, but eggs do not sensibly resemble conspecifics, so it is not clear which sensible stimulus provokes the parents to care for eggs (Reimarus 1762, p. 66, Jaynes and Woodward 1974b). Moreover, birds build their nests long before they lay their eggs and before their young are born, i.e., before their eggs are *visible*. Hence, sensible pleasure and pain received from *external* stimuli does not suffice to explain animal behavior. To explain the parental care that birds exhibit, we must postulate an innate *Kunsttrieb* that leads them to build nests and to take care of their young (Reimarus 1762, pp. 65–70).

More generally, Reimarus believed that external stimuli that elicit pleasure or pain cannot explain any instinctive behavior of animals (ibid., pp. 116–117). While performing instinctive behavior, e.g., a spider weaving its web in order to catch prey, animals always choose the *most perfect* means to accomplish their end. Animals may, through external stimuli, represent some end as pleasurable. However, the capacity and skill to choose the most perfect means to accomplish this end is not provided by this stimulus (ibid., p. 88). To explain this capacity, Reimarus postulated innate skill drives that govern the artful instinctive behavior of animals. These skill drives were not similar to drive concepts such as hunger or a sex drive (Reimarus 1762, p. 114) Hunger, for example, leads to a sensation that drives animals to seek food, but it cannot explain the complicated ways in which animals procure food, e.g., crows that crack walnuts by dropping them from a certain height. Such behavior requires the postulation of drives that govern artful behavior. To conclude: pleasure, pain, and desires do not, contrary to what Buffon believed, suffice to fully explain animal behavior. To explain animal behavior, we must posit innate and unlearned *Kunsttriebe*.

### Reimarus’ justification of positing instincts

In the previous sections we have already distilled several reasons for why Reimarus postulated the existence of animal instincts. These include: (i) the hypothesis of animal instincts was in harmony with the idea of divine creation (theological reason), and (ii) animal behavior cannot be explained mechanically (thesis on the explanatory scope of mechanism). However, the idea that animal behavior cannot be explained mechanically does not necessitate the hypothesis of animal instinct. After all, we could accept this premise and argue that animals have psychological faculties that resemble human psychological faculties (the mentalist hypothesis). To understand why Reimarus rejected the mentalist hypothesis, we must take a closer look at (iii) the empirical evidence Reimarus provided for his theory. Finally, (iv) we will look at the theoretical virtues that can be invoked to justify the hypothesis of animal instincts.

The empirical evidence that Reimarus supplied to support his theory of instinct was manifold. We have already seen that Reimarus appealed to instincts to explain the perfect adaptedness of animal behavior to the environment, as well as the fact that animals choose the most perfect means to achieve certain ends. He also argued that many animals were able to perform complex behavior right after they were born, i.e., before they were able to *learn* this behavior through experience (see also Richards 1987, p. 30). For example, a spider weaves its web in order to catch prey before it was able to learn this behavior (Reimarus 1762, pp. 90–91). Moreover, the behavior patterns that animals exhibit were often taken to be *invariable* and *uniform* (Reimarus 1762, p. 93), which suggested that they did not perform their actions through human-like psychological faculties, such as reason, the latter being responsible for *variable* behavior in response to identical stimuli. All this evidence supported the idea that animal behavior was largely determined by *innate* and *unlearned* instincts. Reimarus did, of course, acknowledge that animal behavior can sometimes be variable. He allowed for the possibility that animals learned through experience, but like Buffon he argued that this learning could be accounted for by association or instrumental conditioning. For this reason, the learning of animals did not necessitate us ascribing reason to them. As examples of learning by association, Reimarus mentioned a horse that enters a hostel where it has received food before, dogs that are afraid of sticks with which they have been beaten, and so forth, all of which are actions which Reimarus explained in terms of the imagination of animals and not reason (Reimarus 1762, p. 20. For details, see van den Berg 2018a, p. 5). Moreover, Reimarus also argued that instincts do not fully determine animal behavior so that there is no room for variable behavior (Reimarus 1762, pp. 172–173). Animal instincts prescribe general patterns for behavior, but behavior may still vary in different circumstances. Thus, for example, instinct prescribes a general model for the building of bird’s nest, but depending on the circumstances different nests may of course be constructed differently.

Since Reimarus took his theory to fit the empirical data of animal behavior, we can conclude that he appealed to the theoretical virtue of *evidential accuracy* to support his theory (Keas 2018). What other theoretical virtues did Reimarus appeal to in support of his theory? Like Buffon’s mechanical theory, Reimarus’ theory of animal instinct seems to be shaped by considerations concerning unification, even though Reimarus does not explicitly mention this virtue. In Chapter 7 of his *Triebe*, Reimarus provides a classification of all instincts (*Kunsttriebe*). He distinguishes ten classes of instincts and 57 instincts in total. The instincts that he mentions are general in scope and are meant to explain the behavior of a manifold of animals. For example, he mentions the instinct to switch from environment during the turn of the season, and notes that this instinct is applicable to birds, four-footed animals, insects and fish (Reimarus 1762, p. 141). In addition, he mentions the capacity of animals to recognize their natural enemies, which ones again applies to a great manifold of animals (ibid., p. 143). Hence, Reimarus’ intended to construct a scheme of animal drives and instincts that allowed him to provide a unified explanation of as many animal behaviors as possible.

To what extent did Reimarus appeal to simplicity when explaining all animal behavior in terms of innate instincts? The answer is that Reimarus uses his own specific notion of simplicity to argue for his theory of instinct. The notion of simplicity that Reimarus employs is not, however, identical to Ockham’s Razor or to the notion of simplicity that we have discussed earlier in the paper. Nevertheless, it is, I shall argue, a notion that comes close to simplicity.

Reimarus refers to a notion of thrift (*Sparsamkeit*) when arguing for his theory of instinct. He argues that the great artificer (God) uses thrift when creating animals. The fact that God creates animals in this way proves he is a great artificer (Reimarus 1762, p. 243). Like other good artificers, who use little forces to lift heavy loads, God endows animals with lower psychological faculties that allow them to perform capacities for which humans require higher psychological faculties (such as reason). The attempt to explain animal behavior in terms of instincts is thus thrifty or parsimonious, insofar as complex behavior is explained in terms of lower psychological faculties. The notion of thrift is not identical to the notion of simplicity that we have discussed earlier in the paper. Nevertheless, it comes close to the notion of simplicity insofar as Reimarus argues that God favors simple designs: God used the simplest design of animals, ensuring that they can perform complex behavior by employing lower psychological mechanisms, and hence we must favor explanations of animal behavior in terms of lower psychological faculties

How should we understand the distinction between lower and higher psychological faculties? This distinction comes from Wolff. He defined lower psychological faculties, such as the senses and the imagination, as lower faculties (*Facultatis cognoscendi pars inferior*) by which we compare *obscure* and *confused* ideas and notions. The higher psychological faculties (*Facultatis cognoscendi pars superior*), such as attention, reflection and the intellect, are those by which we acquire *distinct* ideas and notions. (Wolff 1968, p. 33). Thus, the psychological scheme that distinguishes between obscure and confused ideas and clear and distinct ideas provided the basis for distinguishing between higher and lower psychological faculties. Insofar as Reimarus only ascribes lower faculties of cognition to animals, animals, according to Reimarus, only have obscure and confused representations, and not clear and distinct representations (see for more discussion of this topic van den Berg 2018a).

To conclude: although Reimarus’ appeal to thrift can be seen as an appeal to simplicity, his appeal to thrift is fundamentally different from an appeal to Ockham’s Razor. Reimarus does not appeal to the number of kinds of entities postulated by the theory (ontological simplicity) or to the number and conciseness of the theory’s basic principles (syntactic simplicity) (Baker 2016). Rather, he refers to explanations in terms of lower psychological faculties in the sense of Wolff.

Finally, Reimarus did not appeal to considerations concerning the understanding of theory (UT) to support his position. However, since he thought his theory provided the best explanation of animal behavior it seems obvious that he took his theory to provide understanding of nature (UP).

Let us summarize the reasons Reimarus provided for his theory of instinct: Reimarus argued (i) that the appeal to innate and purposive instincts fits the idea that nature and organisms are divinely created (theological reason), (ii) that animal behavior is mechanically inexplicable and must be explained by appeal to instincts (thesis on the explanatory scope of mechanism), (iii) that the appeal to divinely created and purposive instincts explains (a) the perfect adaptedness of animal behavior to the environment, (b) the fact that animals choose the most perfect means to achieve certain ends, and (c) the fact that many complex animal behavior is uniform and exhibited before animals are capable to learn anything (thus the theory of animal instincts fits the empirical data and Reimarus appealed to the theoretical virtue of evidential accuracy), (iv) the theoretical virtue of unification, (v) a peculiar notion of thrift that comes close to the theoretical virtue of simplicity but is not identical to it, and (vi) the notion of understanding of nature (UP).

## Leroy and Condillac

Although Reimarus rejected Buffon’s claim that the behavior of animals can be explained mechanically, both authors denied reason to animals. In the late eighteenth-century, so-called sensationalist authors (Richards 1979), such as Charles-Georges Leroy (1723–1789) and Etienne Bonnot de Condillac (1714–1780), went a step further and argued that animals did have reason. According to these authors, animals and humans did not differ in kind but only in degree, even though Condillac thought that the key difference between animals and humans was language (Wolfe 2017, p. 162).^9^

Leroy, born in 1723, was a Ranger of Versailles and Marly and the author of the article on instinct in the *Encyclopedie* (Renck and Servais 2005). He wrote a tract entitled *Lettres sur les animaux* of 1768 (hereafter: *Lettres*), in which he aimed to prove on empirical grounds that animals have reason. The tract was published anonymously: Leroy felt the need to disguise the authorship of the *Lettres*, and presented them as the writings of an author from Nuremberg (ibid). The observations of Leroy were partly intended to disprove both Buffon’s mechanism and Reimarus’ theory of instinct. In the following, I will make use of the English translation of Leroy’s works, contained in Leroy (1870).

Leroy is often portrayed as a thorough observational naturalist. Philip Howard Gray noted that Leroy was probably ignored by historians because there was little philosophical content in his work (Gray 1968, pp. 377–379). Marion Thomas has sketched the historical context of Leroy’s *Lettres*, arguing that Leroy’s works can be read against the background of the opposition between cabinet naturalist, exemplified by Buffon, and field naturalists, exemplified by Joseph de Tournefort. Leroy, who stressed the importance of observations of animals conducted by hunters, can be situated in the tradition of field naturalists (Thomas 2008). The most extensive introduction to Leroy’s *Lettres* and his other writings, as well as much biographical information on Leroy, can be found in (Servais and Renk 2005), who stress the importance Leroy placed on animal observations, and compare Leroy’s style of studying animals to those of modern ethologists. Finally, Bourdin has written an article on Leroy’s anthropomorphism (Bourdin 2010), whereas Robert Richards has placed Leroy within the sensationalist tradition, noting, like others, Condillac’s influence on Leroy (Richards 1979, pp. 89–92, 99–100). In the following, I will first present the basics of Leroy’s theory, taking into account the views of Condillac when appropriate. I will then, secondly, discuss which theoretical virtues influenced Leroy and Condillac’s theory.

### Leroy the naturalist: observing animals

Leroy took the aim of his treatise to be the following: to investigate how by repeated sensations animals rise from mere instinct to intelligence (Leroy 1870, p. 10). Intelligent behavior of animals was taken to be manifest when the animal performed variable behavior as a result of experience. The naturalist, according to Leroy, should thus not merely study the structure, character and way of life of animals, but should focus on those circumstances which force an animal to *modify* its behavior. By observing animals in all ages and situations of life, we come to know how experience modifies the behavior of animals and can thus judge correctly concerning the degree of intelligence of animals (Leroy 1870, pp. 11–13).

Leroy’s stress on the modification of animal behavior caused him to pay special attention to the development of behavior during an animal’s life, i.e., to the *ontogeny* of behavior. It also led Leroy to deny, arguing against Buffon and Reimarus, that animal behavior is highly uniform. For example, Leroy described in detail how young wolves learn to hunt and procure food for themselves (Leroy 1870, pp. 18–20). After spending the first two months of their life in the den, young wolves follow their mother in search of prey and learn to chase and consume prey. Through the “habitual practice of rapine”, young wolves learn new ideas, such as the places where game retires, and gradually learn to satisfy their wants. After eight or 9 months, the mother leaves the litter, after which many young wolves need to learn to hunt for prey by themselves. In this period, they often first search for dead animals, and they only slowly develop the strength or the skill to hunt down living animals. All of this showed that wolves learn the capacity to hunt through individual experiences and that hunting behavior was not innate.

Next to focusing on the ontogeny of behavior, Leroy described how the interactions with man modified the behavior of animals (Leroy 1870, pp. 20–25). In places where the wants of animals clash with those of humans, the animal enters into an artificial state of existence that resembles civilization. In this state, the animal modifies its behavior in accordance with its wants and needs. For example, wolves that have been hunted will resist attacking a flock if they recall the impression of a shepherd and his dog. The experience of being hunted by man thus makes the wolf cautious. This type of learning, Leroy claimed, demonstrated the wolf’s intelligence.

On the basis of these and similar empirical studies Leroy concluded against Buffon and Reimarus that animals are *perfectible*, i.e., they have an aptitude for improvement (ibid., p. 34). When young and undisturbed, animals perform regular almost machine-like actions. However, when they are disturbed by man, i.e., when confronted with the fear of pain or death, or when they enter into social relationships with one another, they become skillful, clear-sighted and resourceful. The idea that animal behavior was uniform and imperfectible, which underlay both Buffon’s mechanical account of animal behavior and Reimarus’ theory of instinct, was thus found to be empirically false (ibid., pp. 33–34). In short: Leroy thought that the idea that animals have reason fits the empirical data on animal behavior, i.e., the fact that animal behavior is perfectible, and thus appealed to the theoretical virtue of evidential accuracy to support this theory.

### Why did Condillac and Leroy accept mentalism?

Within our discussion of the *Histoire Naturelle*, we have seen that Buffon valued the theoretical virtue of scientific understanding (both in the sense of UT and UP). In this section, I will show that Leroy and Condillac also valued this theoretical virtue (both in the sense of UT and UP). This virtue led Leroy, like Buffon, to study animals that were familiar to him. It also led both Leroy and Condillac, unlike Buffon, to reject mechanistic explanations of animal behavior and to favor explanations of such behavior that make use of mentalistic notions. Hence, the fact that explanations are “simple for us” (see Heyes 1998, who considers “simplicity for us” as an argumentative device in animal research) or easy to understand was historically invoked as a reason for adopting mentalist theories.

Leroy often studied animals that were familiar to him and hunters. In his dedicatory letter, Leroy remarked that “none but a sportsman can fully appreciate the intelligence of animals. To know them thoroughly, you must have associated with them” (Leroy 1870, pp. 1–2). The naturalist must thus have a thorough personal acquaintance with animals, and must, moreover, study them “in their daily conduct” with which we are familiar (ibid). Moreover, we should study those animals with which we have affinity, which implied, for example, that the study of insects was unsuitable to understanding animal intelligence (Leroy 1870, p. 2). For Leroy, *only* animals that we are familiar with and that are *similar to* humans are appropriate to study animal intelligence. The reason for these claims, as we shall also see below, is that the conduct of these animals is easy to observe and *understand*: we can take ourselves as a model for understanding animal behavior and infer the causes of this behavior by analogy. In my view, Leroy appealed to two senses of scientific understanding: (i) he appealed to the facts that *theories* that explain animal behavior in terms of mentalistic notions, e.g., by attributing human-like reason to animals, are easy to understand because humans have experience with explanations of human behavior in terms of human psychological faculties (UT), and (ii) he thought that explanations of animal behavior in terms of mentalistic notions provide the best scientific explanation of animal behavior and thus provide us with *understanding of nature* (UP).

Condillac and Leroy frequently compared the behavior of animals to the behavior of adult rational humans. For example, in the first chapter of his *Traité*, Condillac argues by analogy that since animals act similarly to humans, they must be sentient beings with thoughts and feelings. It is unreasonable to reject this analogy, Condillac argues, since we adopt the same type of reasoning when attributing perception and thoughts to *other* human beings (Condillac 1984, p. 12). In his *Lettres*, Leroy similarly argues that we are justified in attributing thoughts, feelings and mind to other people based on an analogy with ourselves, and hence it is justified to adopt the same procedure with animals (Leroy 1870, pp. 9; 87–88). On the basis of such arguments, Leroy adopts as a methodological rule that it is “only by reflecting upon our own sensations that we can form a judgment of the animate beings which surround us” (ibid., p. 180). Here, we see how the analogy between rational humans and animals frames research into animals. Not surprisingly, this analogy gives rise to a much more anthropomorphic assessment of animal behavior than that given by Buffon and Reimarus.

Why did Condillac and Leroy value the man-animal analogy so highly? The answer seems to be that the man-animal analogy allows us to better *understand* animal behavior than other analogies (both in the sense of UT and UP). Leroy makes this point explicitly when he remarks that by employing this analogy, we reason from truths that we are absolutely certain of, i.e., our knowledge of our own perceptions, thoughts, etc. We have certain knowledge of ourselves, and hence it is appropriate to use this knowledge, by analogy, to understand the psychology of animals (Leroy 1870, pp. 86–87). On these grounds, Leroy argues that his analogy is better than other analogies, such as the machine-animal analogy (Leroy 1870, pp. 86–88).

In other words, we must use ourselves (man) as a *model* for explaining animal behavior because we are intimately familiar with ourselves. In my view, this claim is based on the idea that theories that use man as a model to explain animal behavior are easier to understand as a theory (UT) and provide proper explanations of nature (UP). The fact that we are intimately familiar with ourselves makes us understand theories that explain animal behavior in terms of human psychological states better. Hence, Leroy both thought that scientific theories that employ mentalistic terminology are better to understand than alternative theories and that mentalistic explanations yield a better explanation and thus understanding of nature. Finally, both Condillac and Leroy prefer the man-animal analogy because they do not think that mechanical explanations of animal behavior yield scientific understanding, again both in the sense of (a) understanding of theory (UT) and (b) understanding of nature (UP). On the contrary, they take such mechanical explanations to be utterly incomprehensible. In the following, we will take a closer look at this argument.

In Chapters 3 and 4 of his *Traité*, Condillac discusses Buffon’s mechanical explanations of animal behavior. As we have seen in Sect. 2, Buffon attempted to mechanically explain the actions of higher animals in terms of the action of the inner sense (the brain). This explanation is rejected by Condillac because it is *incomprehensible* (Condillac 1984, pp. 18–19. Cf. pp. 13, 14–15, for further remarks on the incomprehensibility of mechanism). In a similar vein, Leroy declares that all mechanical theories that explain animal behavior in terms of “material sensations” or “material memory” are “utterly beyond my comprehension” (Leroy 1870, p. 96). Here, the claim seems to be that mechanical explanations of animal behavior are *theories* that are deemed to be incomprehensible.

Why were mechanist theories of animal behavior deemed incomprehensible? One answer is that Condillac and Leroy took Buffon’s mechanism to be arbitrary and hypothetical, i.e., it did not provide understanding of nature. In his *Treatise on Sensations*, Condillac took Buffon’s claim that the nerves, stretched cords capable of “perturbation” and “vibration”, are the cause of sensation and memory to be “completely imaginary” (Condillac 1982, p. 373). The reason is that we do not know “the essence of the mind, the mechanism of the eye, the ear, and the brain” (Ibid., p. 375). According to Condillac, hypothesis about such mechanisms, as proposed by Buffon, are unsupported by empirical data and hence worthless. Hence, the mechanical theory of Buffon lacks empirical support and is not well-grounded by the data, and therefore does not provide understanding of nature. Here, Condillac and Leroy thus once again refer to the evidential accuracy of theories, and argue that Buffon’s mechanical theory does not satisfy this theoretical virtue.

Another empirical argument against mechanism was that mechanist explanations of animal behavior cannot explain the *modifiability* of animal behavior. This was once again a reason to argue that the mechanical theory did not satisfy the virtue of evidential accuracy. The modifiability of animal behavior, resulting in behavior that is *adapted* to changing circumstances, was necessary for their survival. This type of behavior, Condillac and Leroy thought, cannot be explained mechanically, since they took behavior governed by mechanisms to be completely uniform: the same stimulus will always result in the same response. The same argument was leveled against Reimarus’ theory of instinct: instincts cannot explain modified behavior because they result in uniform behavior. In Chapter IV of his *Traité*, Condillac presents this argument by noting that, on the assumption that animals are purely material, we cannot explain how animals “ensure their conservation” (Condillac 1984, p. 19f). The conservation of animals presupposes that they have understanding (ibid). Similarly, Leroy takes the modification of animal behavior to be a mark of understanding that is the result of learning. He contrasts this type of behavior to behavior governed by mechanical causes:

For instance, we should be wrong in ascribing the alarm excited in most carnivorous animals to a mere mechanical impression. The shaking of a leaf rouses in a young wolf only a simple movement of curiosity; but an experienced wolf, who has known this fluttering precede the appearance of a man, takes fright at it with reason, because he has perceived the connection of the two phenomena. When these judgments have been repeatedly formed, and by this repetition the actions to which they lead have become habitual, the rapidity with which the action follows the judgment gives it a mechanical character; but a little reflection enables us to recognize the steps which led to it, and refer it to its true cause. (Leroy 1870, pp. 23–24)

The anthropomorphic explanation of the wolf’s behavior is superior to mechanical explanations because it allows us to explain the modifiability of the wolf’s behavior. To put the point differently: learning requires modifiability (different responses to same stimuli) and mechanism cannot explain modifiability.

Leroy and Condillac explained the modifiability of animal behavior in terms of the animal having intelligence or reason. How did they account for instances of relatively uniform behavior, such as the types of unlearned behavior that Reimarus’ theory of instinct was supposed to explain? As Richards has explained, a common attempt to explain this uniform behavior was through a use-inheritance theory, according to which “long practiced habits gradually molded the heritable structures of behavior and anatomy in a species (Richards 1979, pp. 100–101). Leroy also adopted this theory. Thus, for example, Leroy remarks that “when for many generations the habit of retrieving has been enforced upon dogs, it becomes natural to the race, and will continue to show itself for some time without any particular cultivation”. (Leroy 1870, p. 92). Hence, the uniform behavior of animals resulted from long-standing *habits* that ultimately became heritable.

So why did Leroy and Condillac accept mentalism? They argued that (i) their theory best explained the empirical data (evidential accuracy), (ii) that mechanism and theories of instinct could not explain the empirical data (negative claim concerning the evidential accuracy of alternative theories), (iii) that mentalism provided *scientific understanding* in the following two senses: (a) as a *theory* mentalism is more understandable than other theories such as mechanism (UT), and (b) mentalism provided the best explanation of animal behavior and thus yielded the best *understanding of nature* (UP). The idea behind the claims concerning scientific understanding was that we must use human cognition as a model for understanding animal behavior. Because we are intimately familiar with such a model, we must understand animal behavior on the basis of human cognition.

## Theoretical virtues: a comparison

In the previous sections we have specified the grounds that shaped the views of Buffon, Reimarus, and Leroy and Condillac on animal cognition. These grounds ranged from general metaphysical reasons, theological reasons, moral reasons, to particular theoretical virtues such as simplicity and unification. It is clear that Buffon, Reimarus, and Leroy and Condillac all thought that the explanatory models adopted by their opponents were based on false premises. Thus, for example, Reimarus’ appeal to the teleological structure of nature was inadmissible in a mechanist scheme espoused by Buffon.

Besides general principles informing their explanatory models, the authors we have discussed also all appealed to different theoretical virtues to support their theories. Below I provide a table of the theoretical virtues that the different authors appeal to. In this table, UT stands for the understanding of a theory, UP stands for the understanding of a phenomenon or of nature, and I distinguish the theoretical virtue of simplicity from Reimarus’ notion of thrift.

**Table:** 

	Evidential Accuracy	Causal Adequacy	Simplicity	Unification	UT	UP	Thrift
Buffon	X	X	X	X		X	
Reimarus	X			X		X	X
Leroy/Condillac	X				X	X	

The appeal to different theoretical virtues elucidates that the different authors justified their theories in different manners. This fact partly explains why the different authors adopted their respective positions. It is noteworthy, for example, that Leroy and Condillac referred to scientific understanding to justify their position, both in the sense of the understanding of theory (UT) and the understanding of nature (UP). The appeal to scientific understanding of theory (UT) is a distinctive and unique element in the justification of the sensationalist position.

There are two theoretical virtues that all naturalists appealed to: evidential accuracy and the understanding of a phenomenon (UP), which is the understanding that results from providing an adequate explanation of a phenomenon. It is instructive that these virtues are universally appealed to, as it indicates their importance for eighteenth-century naturalists. In our discussion of logical perfections or theoretical virtues in the eighteenth century, we likewise found that almost all authors appealed to evidential accuracy. Hence, there seemed to be widespread consensus among eighteenth-century philosophers and scientists that theories of natural science should fit the data and that one must provide adequate explanations of the data.

Although all authors appealed to evidential accuracy, the role of experience within theory formation and justification was somewhat problematic. All authors agreed that the theory should fit experience. In addition, all authors agreed that theories of animal behavior should explain both uniform and variable animal behavior. However, different authors stressed different observations to support their respective theories. Thus, Buffon and Reimarus stressed observations that support the uniformity and imperfectability of animal behavior, which they took to support mechanical theories and theories that posit instincts, and constructed their theories around these observations. By contrast, Leroy and Condillac stressed observations that supported the idea that animal behavior is perfectible and variable, such as observations concerning the ontogeny of animal behavior, which they took to support their sensationalist and mentalist theories. Hence, different authors stressed different aspects of experience and made use of observations in a somewhat opportunistic manner. In short: although the theoretical virtue of evidential accuracy was widely accepted in the eighteenth century, there were no explicit or systematic guidelines or procedures in place on how to use experience to decide between different competing theories.

The other theoretical virtues also do not force a choice between the three theories within my case study. This is because (i) the naturalists refer to different theoretical virtues to justify their positions and presumably rank these virtues differently. Since there is (as yet) no algorithm to apply and weigh the criteria, the different choice of the virtues and the different ranking of the virtues means there is no universally accepted decision procedure for theory choice (see the classic work of Kuhn 1977 for this point). In addition, (ii) different authors could appeal to the same virtues to justify their theory. For example, both Buffon’s mechanism and Reimarus’ theory of instinct could appeal to the notion of unification for support. This seems to be a more general possibility: different theories can satisfy the same virtues, and in such cases the theoretical virtues seem incapable of forcing a choice between such theories.

From a historical perspective, which we adopted in this paper, it is not surprising that the different naturalists appealed to different and similar theoretical virtues to justify their positions. As we described in Sect. 2, theoretical virtues (or logical perfections) in the eighteenth century were conceptualized as perfections that a good scientific theory must satisfy. Hence, it is to be expected that naturalists appealed to these virtues to justify their positions. The logical perfections were not, as we have also seen, explicitly conceptualized as guiding theory choice. However, in our discussion of Buffon, Reimarus, Leroy and Condillac, it became clear that the different naturalists certainly appealed to theoretical virtues to justify their preference for their own position over and above alternatives (see, e.g., the criticism of mechanism in Reimarus, or the criticism of mechanism in the mentalist position of Leroy and Condillac). Hence, the theoretical virtues or logical perfections in the eighteenth century gave criteria for what counts as a proper scientific theory, and although they were not explicitly conceptualized as guiding theory choice, they factually were invoked to facilitate the choice between different scientific theories. There is thus a lot of continuity between our present-day conception of theoretical virtues and the role theoretical virtues played in the eighteenth-century life sciences. Finally, we may note that the naturalists often referred to the same virtues, such as evidential accuracy and unification, in order to justify their theories. This is also not surprising given that these virtues define what a proper scientific theory looks like: philosophers and scientists would have felt the need to show that theories exemplified the different virtues as much as possible. Our analysis of theoretical virtues in the eighteenth century and of the use of these virtues in eighteenth-century debates on animal cognition thus explains why theoretical virtues were so often invoked in debates within the life sciences,

## Conclusion

In this paper, I have given an analysis of the role and status of theoretical virtues in eighteenth-century philosophy and the life sciences. I have first given a general account of how theoretical virtues were conceptualized in eighteenth-century German and French philosophy and science. Subsequently, I have studied how these theoretical virtues were used in the justification of eighteenth-century theories of animal cognition. Through my analysis, we have obtained historical understanding of how theoretical virtues were understood in the eighteenth century and have obtained a more complete understanding of the different justificatory strategies in eighteenth-century debate on animal cognition.

## Footnotes

1 For a classic account of simplicity or parsimony, see Sober (1988).

2 Discussing Buffon’s Newtonianism and his account of organic particles, Gaukroger, like Reill 2005, has presented Buffon as a fierce critic of mechanism (2010, pp. 344–345; pp. 363–383). It should be noted, however, that Buffon’s account of animal behavior is strongly mechanistic, and that he often describes himself as a mechanist. My account is not fundamentally at odds with that of Gaukroger and Reil, who contrast Buffon’s theories to seventeenth-century conceptions of mechanism. For it is important to understand that the concepts of mechanism and mechanical explanation shifted in the eighteenth-century to accommodate, among others, the findings of Newton. See McLaughlin (1990); van den Berg (2014).

3 Buffon explicitly identifies the brain with the internal sense: “I apprehend, therefore, that, in the animal, the action of objects on the senses produces another on the brain, which I consider as a general internal sense […]” (Buffon 1785b, p. 224). On the history of the complicated notion of internal sense, see Azouvi (1984).

4 I wish to thank an anonymous referee for stressing this point.

5 See Roger (1997b), Sloan (1992), Mensch (2013), van den Berg (2018b).

6 Gaukroger (2014, p. 220) claims that for Buffon *only* human interests determine how we describe nature. He also argues that according to Buffon there is no natural system and that the criterion of interbreeding, which Buffon used as a criterion of species inclusion, is a purely economical principle (2010, pp. 194–196). Human interest are indeed important to Buffon. However, Buffon also describes his method as one “which sustains the very order of things” (Buffon 1976, p. 172). This suggests that Sloan is correct: Buffon took his method to be superior to others since it allows us to better grasp the order of nature. Further evidence for Sloan’s reading is contained in Buffon’s natural history of the dog. There he presents experiments which showed that dogs do not interbreed with wolves and foxes. This proved that they are distinct species, that the wolf, the fox, and the dog “are very different in their nature”, that the dog “derives not his origin from the wolf or the fox”, and that Linnaeus, who regarded foxes and wolves as wild dogs, did not sufficiently study nature (Buffon 1785c, p. 27). Clearly, then, the criterion of interbreeding is not *merely* economical: it informs us about the order of nature.

7 On the similarities between Reimarus and Kant, see van den Berg (2013) and van den Berg (2018a).

8 I will develop this argument in detail below.

9 We should note that the three positions on animal behavior and intelligence that we have discerned (mechanism, instinct, and mentalism) were also combined in original ways in the eighteenth century. In his *The Philosophy of Natural History* (1790), the naturalist William Smellie, rejecting pure mechanism and attributing some form of reason to animals, adopted a synthesis of an instinct theory of animal behavior and a form of mentalism.  Smellie argued that no strict distinction between instinctive and rational motives existed. According to Smellie, the “reasoning faculty itself is a necessary result of instinct” (1790, p. 145). Smellie distinguished between three types of instincts: (1) pure instincts, which independently of experience produce certain actions when certain objects are presented to animals (e.g., the sucking of a baby) (1790, pp. 145–150), (2) instincts which can accommodate themselves to peculiar circumstances and situations (e.g., ostriches that depending on the heat sit on the eggs) (1790, pp. 150–152). These instincts thus exhibit a certain amount of adaptation to (changing) external circumstances. Finally (3), Smellie described instincts which are improveable by experience  and observation, such as many human instincts (1790, pp. 152–154). These different types of instinct can be interpreted as ranging from more simple instinctive behavior to more complex instinctive and intelligent behavior. Smellie concluded that “all the modified, compounded, or extended passions and propensities of the human mind, may be traced back to their original instincts” (1790, p. 154). Another author who came close to a synthesis between an instinct theory of animal behavior and mentalism was James Hutton, who in his *Investigation of the Principles of Knowledge* (1794) attributes reason, thought, and judgment to animals, while also arguing that animals are governed by regular instinctive behavior (1794, p. 514). As Hutton puts it: “the first instinctive action of the animal is thus the same, in principle, as the last action of his life; it changes not its nature, however it may be diversified in the circumstances or natural motives by which it is conducted” (Ibid.). This leads Hutton to argue that animals are governed by a determinate natural order and lack wisdom, in the sense of a power of choosing the best end of an action or the best means for happiness (Hutton 1794, p. 516). It is man who possesses wisdom, and man is thus distinguished from animals by the fact that man acts as “a superior being, in forming abstract general opinions, conceiving system, and designing with intelligence” (Hutton 1794, p. 516).

## References

[CR1] Allen C, Bekoff M (1997). Species of Mind. The philosophy and biology of cognitive ethology.

[CR2] Azouvi F (1984). Quelques Jalons Dans La Préhistoire Des Sensations Internes. Revue de Synthese.

[CR3] Baker, A. (2016). Simplicity. In E. N. Zalta (Ed.), *The Stanford encyclopedia of philosophy* (Winter 2016 Edition). https://plato.stanford.edu/archives/win2016/entries/simplicity/.

[CR4] Bourdin JC (2010). L’anthropomorphisme de Charles-Georges Leroy. Chasseur et Philosophe. Dix-huitième siècle.

[CR5] Buffon, G. L. (1785a). *Natural history, general and particular*. (Vol. 2, W. Smellie, Trans.). London: Strahan and Cadell.

[CR6] Buffon, G. L. (1785b). *Natural history, general and particular*. (Vol. 3, W. Smellie, Trans.). London: Strahan and Cadell.

[CR7] Buffon, G. L. (1785c). *Natural history, general and particular*. (Vol. 4, W. Smellie, Trans.). London: Strahan and Cadell.

[CR8] Buffon GL (1976). The initial discourse to Buffon’s *Histoire Naturelle*: The first complete english translation. Translated by John Lyon. Journal of the History of Biology.

[CR9] Cheung T (2006). Hermann Samuel Reimarus’ Theorie der “Lebensarten” und “Triebe”. Sudhoffs Archiv.

[CR10] Cheung T, Buchenau S, Lo Presti R (2017). Cabanis & the order of interaction. Human & animal cognition in early modern philosophy and medicine.

[CR11] Condillac, E. B. (1809). *The logic of Condillac* (J. Neef, Trans.). Philadelphia Printed.

[CR12] Condillac, E. B. (1982). *Philosophical writings of Etienne Bonnot, Abbé de Condillac* (F. Philipp, Trans.), with the collaboration of Harlan Lane. Hillsdale, NJ: Erlbaum.

[CR13] Condillac EB (1984). Traité des Animaux.

[CR14] De Angelis S, Theis R, Aichele A (2018). Physik. Handbuch christian Wolff.

[CR15] De Jong WR, Betti A (2010). The classical model of science: A millenia-old model of scientific rationality. Synthese.

[CR16] De La Mettrie. J. (1745). *Histoire naturelle de l’ame*. La Haye: Jean Neaulme.

[CR17] De Regt H (2017). Understanding scientific understanding.

[CR18] Duchesneau F, Buchenau S, Lo Presti R (2017). Degrees & forms of sensibility in Haller’s physiology. Human & animal cognition in early modern philosophy and medicine.

[CR19] Falkenburg, B. (2000). *Kants Kosmologie. Die wissenschaftliche Revolution der Naturphilosophie im 18. Jahrhundert*. Frankfurt am Main: Vittorio Klostermann.

[CR20] Gaukroger S (2010). The collapse of mechanism and the rise of sensibility. Science and the shaping of modernity, 1680–1760.

[CR21] Gaukroger S (2014). The natural and the human Science and the shaping of modernity, 1739–1841.

[CR22] Gaukroger S, Buchenau S, Lo Presti R (2017). Anthropological medicine & the naturalization of sensibility. Human & animal cognition in early modern philosophy and medicine.

[CR23] Gray PH (1968). The early animal behaviorists: Prolegomenon to ethology. Isis.

[CR24] Guerrini A (2012). Perrault, Buffon and the natural history of animals. Notes & Records of the Royal Society.

[CR70] Heyes CM (1998). Theory of mind in nonhuman primates. Behavioral and Brain Sciences.

[CR25] Hoquet T (2010). History without time: Buffon’s natural history, as a non-mathematical physique. Isis.

[CR26] Hutton J (1794). An investigation of the principles of knowledge and of the progress of reason, from sense to science and philosophy.

[CR27] Ingensiep HW (1996). Tierseele und tierethische Argumentationen in der deutschen philosophischen Literatur des 18. Jahrhunderts. NTM International Journal of History and Ethics of Natural Sciences, Technology and Medicine.

[CR28] Jaynes J, Woodward W (1974). In the shadow of the enlightenment: I. Reimarus against the epicureans. Journal of the History of the Behavioral Sciences.

[CR29] Jaynes J, Woodward W (1974). In the shadow of the enlightenment: II. Reimarus and his theory of drives. Journal of the History of the Behavioral Sciences.

[CR30] Kant, I. (1998). *Critique of pure reason* (P. Guyer, Trans., A. W. Wood, Ed.). Cambridge: Cambridge University Press.

[CR31] Keas MN (2018). Systematizing the theoretical virtues. Synthese.

[CR32] Kuhn TS, Kuhn TS (1977). Objectivity, value judgment and theory choice. The essential tension.

[CR33] Leroy CG (1870). The intelligence and perfectibility of animals from a philosophic point of view.

[CR34] Lötzsch F, Lötzsch F (1979). Vorbericht des Herausgebers. Hermann Samuel Reimarus Vernunftlehre.

[CR35] Lovejoy AO (1936). The great chain of being: A study of the history of an idea.

[CR36] McLaughlin P (1990). Kant’s critique of teleology in biological explanation: antinomy and teleology.

[CR38] Meier GF (1752). Vernunftlehre.

[CR39] Mensch J (2013). Kant’s organicism. Epigenesis and the development of critical philosophy.

[CR40] Müller-Wille S (2007). Collection and Collation: theory and practice of Linnaean botany. Studies in History and Philosophy of Biological and Biomedical Sciences.

[CR41] Reill PH (2005). Vitalizing nature in the enlightenment.

[CR42] Reimarus H (1762). Allgemeine Betrachtungen über die Triebe der Thiere, hauptsächlich über ihre Kunsttriebe.

[CR43] Reimarus H (1766). Die Vernunftlehre.

[CR44] Reimarus H (1766). Die Vornehmsten Wahrheiten der natürlichen religion.

[CR45] Reimarus H (1772). Die Vornehmsten Wahrheiten der natürlichen religion.

[CR46] Renck JL, Servais V, SIgaut F, Renck J-L, Servais V (2005). Charles-Georges Leroy (1723–1789), une vision large de l’animal. L’intelligence des animaux.

[CR47] Richards RJ (1979). Influence of sensationalist tradition on early theories of the evolution of behavior. Journal of the History of Ideas.

[CR48] Richards RJ (1987). Darwin and the emergence of evolutionary theories of mind and behavior.

[CR49] Riskin J (2016). The restless clock: A history of the centuries-long argument over what makes living things tick.

[CR50] Roger, J. (1997a). *Buffon: A life in natural history*. Ithaca: Cornell University Press (Translation of *Buffon, un philosophe au Jardin du Roy*. Paris: Fayard, 1989).

[CR51] Roger, J. (1997b). *The life sciences in eighteenth*-*century french thought* (R. Ellrich, Trans.). Stanford, CA: Stanford University Press.

[CR52] Sloan P (1976). The Buffon-Linnaeus controversy. Isis.

[CR53] Sloan P, Gayon J (1992). Organic molecules revisited. Buffon 88: Actes du colloque international Paris-Dijon-Montbard.

[CR54] Smellie W (1790). The philosophy of natural history.

[CR55] Sober E (1988). Reconstructing the past: Parsimony, evolution, and inference.

[CR56] System. In *The encyclopedia of Diderot & d’Alembert collaborative translation project* (S. J. Gendzier, Trans.). Ann Arbor: Michigan Publishing, University of Michigan Library, 2009. Web. 26 July 2020. http://hdl.handle.net/2027/spo.did2222.0001.321. Trans. of Systeme, *Encyclopédie ou Dictionnaire raisonné des sciences, des arts et des métiers,* Vol. 15. Paris, 1765.

[CR57] Thomas M (2008). Review of L’intelligence des animaux selon Charles-Georges Leroy (1723–1789). Revue d’histoire des Sciences.

[CR58] Van den Berg H (2013). The Wolffian roots of Kant’s teleology. Studies in the History of Philosophy of Biological and Biomedical Sciences.

[CR59] Van den Berg H (2014). Kant on proper science: Biology in the critical philosophy and the *Opus postumum*.

[CR60] Van den Berg H (2018). A blooming and buzzing confusion: Buffon, Reimarus, and Kant on animal cognition. Studies in History and Philosophy of Biological and Biomedical Sciences.

[CR61] Van den Berg H (2018). Kant and the scope of analogy in the life sciences. Studies in History and Philosophy of Science.

[CR62] Van den Berg, H. & Demarest, B. (forthcoming). Axiomatic natural philosophy and the emergence of biology as a science. *Journal of the History of Biology.* 10.1007/s10739-020-09609-2 PMC7538396 32548749

[CR63] Vanzo A, Garber D, Rutherford D (2015). Christian Wolff and experimental philosophy. Oxford studies in early modern philosophy.

[CR64] Wolfe CT, Buchenau S, Lo Presti R (2017). Boundary crossings: The blurring of the human/animal divide as naturalization of the soul in early modern philosophy. Human & animal cognition in early modern philosophy and medicine.

[CR65] Wolff C (1968). Psychologia empirica.

[CR66] Wolff, C. [1728] (1963). *Preliminary discourse on philosophy in general* (R. J. Blackwell, Trans.). Indianapolis: Bobbs-Merril.

[CR67] Wolff, C. [1751] (2003). *Vernünfftige Gedancken von Gott, der Welt und der Seele des Menschen, auch allen Dingen überhaupt*. Hildesheim: Olms.

[CR68] Wolff, C. [1754] (1978). *Vernünfftige Gedancken von den Kräften des menschlichen Verstandes und ihrem richtigen Gebrauche in Erkäntniss der Wahrheit.* Hildesheim: Olms.

[CR69] Zammito JH (2018). The gestation of German biology: Philosophy and physiology from Stahl to Schelling.

